# Change-detection training and its effects on visual processing skills

**DOI:** 10.1038/s41598-022-15649-x

**Published:** 2022-07-25

**Authors:** Jennifer Truong, Martin Buschkuehl, Rachel N. Smith-Peirce, Audrey A. Carrillo, Aaron R. Seitz, Susanne M. Jaeggi

**Affiliations:** 1 School of Education, University of California-Irvine, Irvine, CA USA; 2 MIND Research Institute, Irvine, CA USA; 3 Department of Psychology, University of California-Riverside, Riverside, CA USA

**Keywords:** Human behaviour, Learning and memory, Sensory processing

## Abstract

Previous cognitive training research with the change-detection paradigm found only sparse effects that went beyond improvements in the training task but stressed an increase in fidelity of internal memory representations. Motivated by the demanding visual processing requirements of change-detection training, we extended this work by focusing on whether training on a change-detection task would improve visual processing skills. Fifty participants were randomly assigned to train on a change-detection task or on a control task for seven sessions. Participants’ visual processing skills were assessed before and after the intervention, focusing on visual search, contrast sensitivity, and contour integration. Our results suggest a general improvement in perceptual skills that was primarily driven by a conjunction search task and to a much lesser extent by a complex visual search task and a contrast sensitivity task. The data from the conjunction search task further suggest a causal link between training and improvements of perceptual as opposed to attentional processes. Since the change-detection paradigm is commonly used to assess working memory capacity, future research needs to investigate how much of its variance is explained by memory performance and how much is explained by perceptual processes.

## Introduction

Detecting visual change in the environment is very important and in its most dramatic case even makes a difference over life and death, for example by detecting threats in the form of predators or oncoming traffic. A popular laboratory paradigm to assess change-detection performance focuses on the capacity of visual short-term memory. It presents participants with an initial set of stimuli such as colored squares^1^ or blocks oriented at different angles^2^. After a brief presentation of these stimuli, followed by a pause, the task requires to determine whether a probe is identical to the initial set or not. By varying the number of stimuli, memory capacity can be assessed. The task has been a preferred means to assess working memory capacity because it is assumed that strategies such as rehearsal only minimally affect performance^1^. It has further been argued that individual differences remain stable across an extended period of time^3^, that change detection represents a stable trait^4,5^, and that change-detection performance correlates well with measures of intelligence^6^. Nonetheless, previous training research has revealed that participants are able to considerably improve their performance in such change-detection tasks over time^2,7,8^. Despite limited evidence that training on change-detection tasks impacts performance in untrained measures^7^, there is accumulating evidence indicating that change-detection training improves the fidelity of internal memory representations^2,9,10^, but see^7^ and furthermore, this literature also points towards an increase in visual working memory capacity^2,7,8,10^, but see^11^.

Although the change-detection task is mostly described as a measure to assess visual memory capacity, the task also requires participants to rapidly process visual stimuli. As such, change-detection training will potentially not only foster visual working memory capacity, but more generally, processes related to processing speed and perceptual learning^12^. Specifically, when using a change-detection task for training where the number of stimuli is adaptively increased while holding the presentation rate constant, an increasingly higher rate of processing is required for successful task completion^7^. It has been argued that higher visual processing speed may be the basis for generally improved visual functions^13–16^. This line of research has focused on training contrast sensitivity using Gabor patches as stimuli and has repeatedly resulted in generalizing effects going beyond the specific training paradigm^16,17^. Such generalizing effects led to the emerging view that perceptual learning does not exclusively lead to improvements of low-level processes, but instead, involves optimization of an extensive array of brain networks^12^. The goal of the present study was to explore whether targeted training on a change-detection task has the potential to improve specific visual perceptual processes, and as such, generalizes beyond improvements in memory representations and memory capacity. For that purpose, we focused on potential transfer to perceptual processes that to our knowledge have not received any attention in this training literature. In a first step to investigate such transfer, we focused on visual figure-ground discrimination, contrast sensitivity, and contour integration.

*Visual figure-ground discrimination* is the concept of being able to discriminate an object from its background^18,19^. Visual search is considered a sub-skill of visual discrimination and refers to the process of detecting a target among distractors^20^. Performance on a visual search task directly depends on visual processing speed, that is, the faster an individual is able to process individual stimuli, the better an individual’s performance. It has also been shown that visual search tasks are malleable, and susceptible to training^21^. Another factor that determines performance on a visual search task is working memory. Several studies have shown considerable similarities between visual search and working memory^22,23^. For example, Emrich et al.^24^ asked their participants to perform a visual search task and a visual working memory task. During both tasks, the authors recorded contralateral delay activity (CDA) levels via an electroencephalogram (EEG). Results showed that the CDA amplitudes were comparable in both tasks, suggesting a similar underlying neural mechanism. Using CDA amplitudes as well, Luria and Vogel^25^ found that an increase of visual search task difficulty corresponded with an increase of CDA amplitudes, indicating an increased reliance on working memory capacity to perform the visual search task. This finding corresponds well with the notion that visual working memory capacity is critical to perform well on visual search tasks due to the need to remember the visual target, compare target information with distractor information, and organize the search array^26,27^. While the reason for any potential transfer to visual search tasks can emerge due to perceptual learning and/or induced changes to the working memory system, the visual search task data will allow us to make inferences whether any effects are based on improved perceptual processes and/or increased search efficiency (see also below).

*Contrast sensitivity* refers to the skill to perceive small differences in luminescence between an object and its background^28^. Often operationalized with Gabor stimuli, contrast sensitivity tasks have often been used as a training vehicle^17^. The data from several studies provide evidence for a link between working memory and contrast features in visual processing. For example, Xing et al.^29^ were able to decode the content of working memory by analyzing the brain activation pattern of the sensory cortices by means of functional Magnetic Resonance Imaging (fMRI). While such decoding has been shown before for orientation features of visual stimuli^30^, Xing et al.^29^ were able to do this for stimuli that differed in contrast. Despite a possible connection between contrast sensitivity and working memory, our operationalization to assess contrast sensitivity did not include a working memory component and therefore, any potential transfer in this task is likely to be deemed of a perceptual nature.

*Contour integration* is based on the Gestalt law of “good continuation”, referring to the preference in viewing an object as something that is smooth and continuous rather than something that is abrupt and disconnected through the integration of local elements to form a global contour^31,32^. Contour integration is a special case of visual grouping, which refers to the idea that the visual system tends to group elements into visual wholes through simple rules^33^. Visual grouping has been found to benefit visual working memory, as illustrated by Li et al.^34^. Their study focused on the grouping effect of illusory contours, which is a phenomenon in contour integration in which a person observes contours that are not physically present^35^. Similar to the contrast sensitivity task, our implementation here did not include a working memory component and consequently, any observed transfer to this untrained task would more likely be attributed to improved perceptual processes.

To test our hypothesis whether training on a change-detection task has a causal impact on visual perception, we conducted an intervention study in which participants trained for seven sessions on a similar change-detection paradigm that we used for training before^7^. In this previous work, participants trained ten sessions for a total of 3000 trials. In order to shorten the study time in terms of days participants are trained and are tested on the outcome measures, we opted for seven days of training but increased the daily training time by 25%. This adjustment resulted in 2800 training trials (a 7% reduction of the original total training time) at the end of the study but allowed a participant to complete the study within a time frame of nine days. In addition, we chose to implement a slightly different training task compared to what we have used before. In particular, this involved including a whole array probe because we assumed it would foster configurational grouping that could be beneficial, especially for contour integration, and we also included a mask to reduce the influence of iconic memory, and we excluded trial feedback to reduce the affordance to develop very task-specific strategies. The intervention was book-ended by assessment sessions that consisted of a conjunction search task, a complex visual search task, a contrast sensitivity task, and a contour integration task. The data from the experimental group was compared to an active control group that was tested on the same measures as the experimental group. We expected to find an overall effect of change-detection training on visual processing skills. We expected the strongest effects in the visual search tasks, given their discussed connection to perceptual processes and working memory. However, we predicted a stronger effect in the conjunction search task due to its similarity with the experimental training task for example regarding the discrete nature of stimuli in both tasks and a related task objective which consisted of identifying a target stimulus among a set of discrete distractors. We hypothesized that such task similarity fosters the applicability of rules acquired during perceptual learning to a novel task^36^. In contrast, we expected a smaller effect in a more complex visual search task because we hypothesized that the discrete nature of the training task would not provide enough affordances to increase performance in a task that uses scenes as stimuli that are substantially larger and more complex. Regarding the contour integration task, we argued before that change-detection training could help participants to become better at configurational grouping, which, if indeed true, could have an especially beneficial effect on its performance^7^.

## Methods

### Participants

Undergraduate students from UC Irvine (UCI) were recruited to participate in the study via email and the UCI Human Subjects Lab Pool between June 2020 and February 2021. All experimental procedures performed in this study were approved by the UCI Undergraduate Research Opportunities Program (UROP) following guidelines by the UCI Institutional Review Board and in accordance with the Declaration of Helsinki. Informed consent was obtained from all individual participants included in the study. A total of 53 students participated in the study. Two participants dropped out due to scheduling conflicts and one participant was excluded due to non-compliance with instructions, leaving a final sample of 50 participants. 25 participants (20.48 years old [SD = 1.19]; 72% women) were randomly assigned to the experimental group and the other 25 participants (20.20 years old [SD = 1.38]; 84% women) were assigned to an active control group. The inclusion criteria required participants to have normal or corrected to normal vision and to not be colorblind. Participants' vision health was assessed by self-report that focused on topics such as current eye correction, last vision test, and participants’ general feeling about their vision (i.e., clarity and pain). Participants were asked to fill out a visual activities questionnaire^37^, and color blindness was assessed through a color discrimination test where they were required to choose appropriate color words that matched a colored object. For their participation, participants received either a $65 gift card or a $20 gift card and course credit.

### Training tasks

#### Experimental group: change-detection training

**Figure 1:**
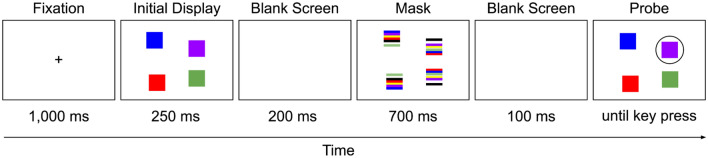
A sample trial of the change-detection training task. Participants were asked to focus on a fixation cross, which was then replaced by a set of colored squares (shown here with set size four). After a blank screen, followed by a mask, followed by another blank screen, a probe of colored squares was presented. Participants had to indicate whether or not the encircled square was of the same color as the one in the initial display.

Participants were asked to train once per day for seven sessions with the change-detection task at home on their own computer. Each training session consisted of 20 rounds, each round comprising 20 trials. A trial started with a fixation cross presented in the center of the screen (Fig. 1). After 1000 ms, the fixation cross was replaced by colored squares that stayed on the screen for 250 ms. After a 200 ms blank screen, a mask was presented for 700 ms, followed by a 100 ms blank screen. The mask consisted of randomly striped squares which appeared in the same locations as the initial stimuli and the colors of the stripes were the same as the ones used for the squares. Next, participants were presented with a probe of colored squares with one square encircled. Participants had to indicate whether the encircled square was of the same color or not as in the initial display. Assuming an eye-to-monitor distance of 50 cm, the array of squares was presented in an imaginary rectangle with a horizontal size of about 12.66° of visual angle and a vertical size of about 9.5° of visual angle. The squares were presented in random locations in every trial with the restrictions that the squares did not appear within an imaginary circle of 2° diameter located in the center of the screen and the center-to-center distance of the squares was at least 2° of visual angle. The squares’ size was about 0.8° × 0.8° of visual angle. The colors used for the squares were black, blue, green, purple, red, white, and yellow. In case of a change-trial (i.e., the cued square was of a different color compared to the initial display), the probe color was randomly selected from this set of colors, excluding the color the square had in the initial display. To account for the variation in participants’ screen sizes and resolutions, they were asked to measure the physical size of their screen in the first training session. After submitting the measurement to the program, the stimuli were adjusted as best as possible to adhere to the indicated degrees of visual angles.

Participants started with a set size of two squares in their first session. The training difficulty of every round was adaptive and depended on participants’ performance on their previous round. If participants achieved an accuracy greater than 85% in a round, the set size would increase by one in the next round. If participants achieved an accuracy of less than 70%, the set size would decrease by one in the next round. If participants scored between 70 and 85%, then the set size would not change in the next round. The starting set size of subsequent sessions was determined by the participants’ last set size in the previous session minus two in order to allow for a brief warm-up period. The minimum set size was two squares, and the maximum set size was 20 squares, but this maximum was not achieved by any of the participants. The dependent measure to quantify training progress was the average set size per session.

#### Active control group

Participants assigned to the active control group were asked to train on a general knowledge task, once per day for seven sessions at home on their own computer. Participants were presented with general knowledge and vocabulary questions and had to select the correct answer out of four provided answer choices within a 15 s time limit. After every response, participants were provided with feedback and incorrectly answered questions were shown again in the next session, offering a learning opportunity. The task consisted of 20 rounds with each round consisting of eight trials. The task was adaptive. If participants scored 75% or better, the level of difficulty of the next round increased by one. If participants scored 50% or less, the level of difficulty of the next round decreased by one. If participants scored between 50 and 75%, the level of difficulty of the next round did not change. The dependent variable to quantify training progress was the average level of difficulty achieved in each session.

A very similar control task has been used before by our lab in other cognitive training work^38,39^. The main rationale of including a knowledge-based task is that it shares only very few if any processes of interest with the experimental task. For example, the presentation rate was not nearly as fast as in the experimental group and the focus of the task was on retrieval of semantic information and not on processing of information that differed on a trial-by-trial basis. In addition, our knowledge-based control task is typically perceived as an actual intervention, and thus, participant expectations have shown to be matched with other cognitive interventions^39^. A minimal overlap of processes of interest and the believability of the control condition to be an actual intervention are both recommended features of control tasks in the cognitive training field^38^.

### Outcome measures

#### Conjunction search

**Figure 2:**
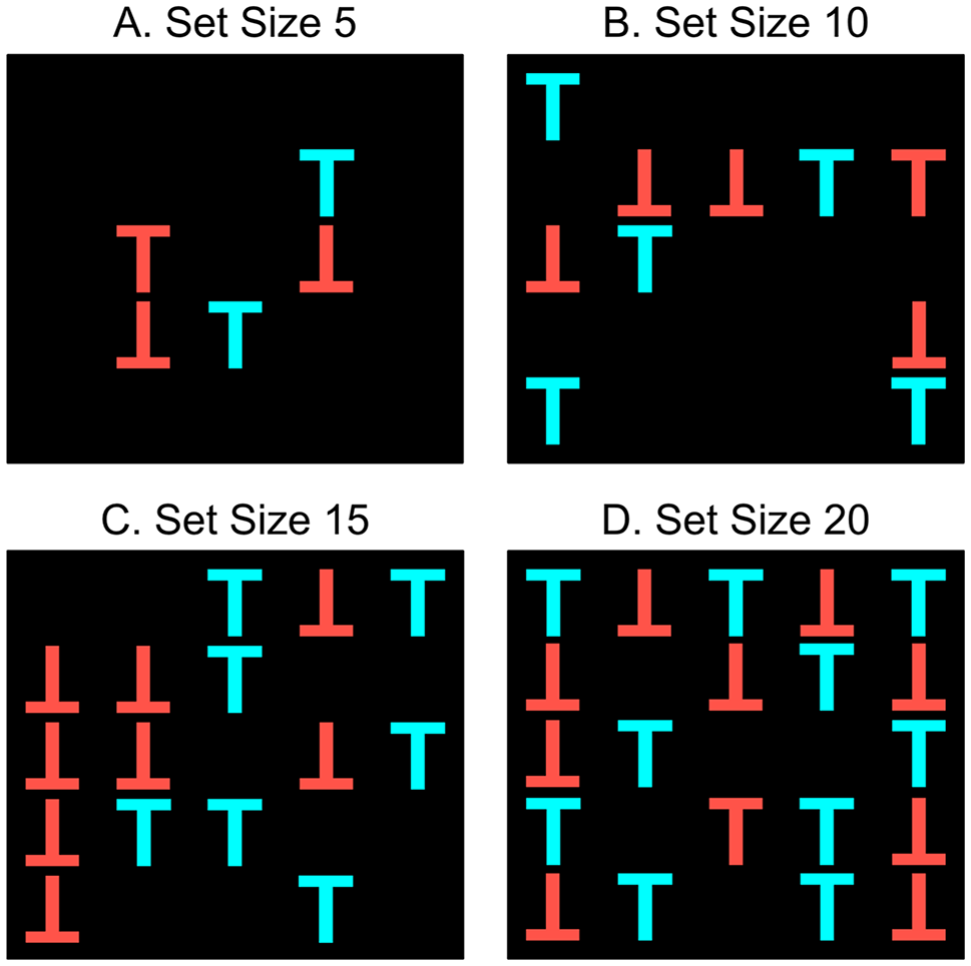
Conjunction search task. Participants were presented with an array of orange letter Ts, orange Ts that were presented upside-down, and blue Ts. The stimuli were presented in set sizes 5, 10, 15, and 20 (Panels A through D). Participants were instructed to look for an orange T and ignore all other stimuli.

Similar to a task used by Stoet^40^, participants were presented with an array of stimuli and asked to find a target among distractors. The target consisted of a regularly presented orange letter T, the distractors of an orange letter T presented upside-down and a regularly presented blue letter T. Participants were presented with 5, 10, 15, or 20 stimuli (= set size) that were with one restriction randomly distributed on a centrally presented, imaginary five by five grid (Fig. 2). The only exception to the random distribution of stimuli within the grid was that the target could not appear in the center location. The two distractor types were as evenly distributed as possible so that the number of upside-down orange Ts and the number of blue Ts differed by maximally one item (e.g., in even set sizes with a target). The set size was randomly selected for each trial as opposed to a blocked presentation. The stimuli had a size of 50 by 50 pixels and the center-to-center distance in the grid was 100 pixels. In contrast to the training task, the size of the stimuli was not adjusted to different screen resolutions and sizes. For each set size, 20 trials were presented and half of them included a target stimulus. A trial started with a fixation cross presented for 2000 ms in the center of the screen, followed by the array of stimuli. Participants were instructed to press the spacebar when they found a target and they were given a maximum of 3000 ms to respond. Participants did not have to press a key if they determined that there was no target on the screen but instead had to wait until the trial timed-out. A response or time-out was followed by a fixation cross presented for 1000 ms, followed by a mask presented for 200 ms. The mask consisted of interleaved orange and blue squares sized 28 by 28 pixels that changed to the opposite color at a rate of 50 ms. Participants were provided with eight practice trials at set sizes 3 and 6, half of them including a target. The dependent variables were the reaction times to correct responses both, across all trials and separately as a function of set size.

#### Complex visual search

This computerized task was based on the well-known ‘Where’s Waldo’ search images^41,42^. Participants were presented with images that were very rich in detail and were instructed to look for ‘Waldo’, a cartoon character with round glasses and a red-white hat. A trial started with the message ‘Get ready for the next image! Press mouse button to start’. After a mouse click, followed by a 500 ms delay, a search image was presented. Instructions stressed to work as quickly as possible without making any errors. Within a time-limit of three minutes, participants had to find the target by clicking on it with the mouse pointer. Participants could click on potential targets as often as they wanted to. After a target was found or the time ran out, the next search image was presented. Through piloting, we created two parallel-versions of the task, each consisting of 12 images, that were comparable in reaction times of finding the target. The dependent variable was the reaction time for correct responses to find the target.

#### Contrast sensitivity

**Figure 3:**
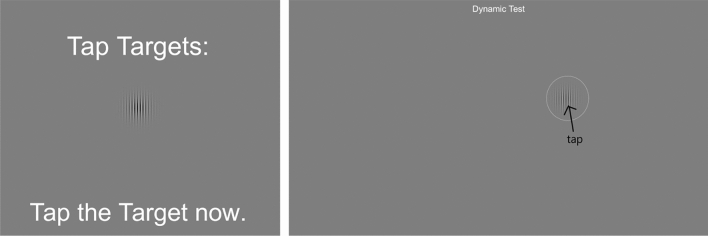
Contrast sensitivity task. Gabors appeared one at a time on the screen against a gray background. Participants were instructed to tap on the patch as quickly as possible. The circle surrounding the Gabor is for illustrational purposes.

This task was based upon low-contrast target detection tasks previously used in studies of visual perceptual learning^43^. Participants were presented with Gabors (mathematically a sine-wave windowed by a Gaussian) shown one at a time at random positions on the screen (Fig. 3). Gabors were presented at 12 cycles per degree (with a standard deviation of 0.75 degrees for the Gaussian window and thus an extent of ~ 2.5° on screen—based on a typical viewing distance of 18″) for up to 4 s each, with a 50 ms ramp to mask the onset. A linear color look-up table was used to ensure proper presentation of the Gabors’ luminance profile and Michelson contrast was calculated. While we cannot guarantee that this leads to perfect rendering of the luminance profile of the Gabors on all devices, these settings are reliable on most modern tablets and smartphones and to whatever extent that there was any distortion of the luminance profile for some participants this would be equated in the pre- and post-test sessions. As it is standard for estimates of contrast sensitivity, the Gabors were presented on a mid-gray background^28^. Participants were instructed to tap on them as soon as they became aware of them. As soon as participants responded, a new Gabor was presented and a 3-down, 1-up staircase was run on the contrast of the Gabors, with approximately 40 trials per run (partially dependent upon the staircase). Thus, subsequent patches were harder to detect. The dependent variable was the contrast threshold (estimated as the average of the last 3 reversals of the staircase) for each participant determined by the staircase procedure.

#### Contour integration

**Figure 4:**
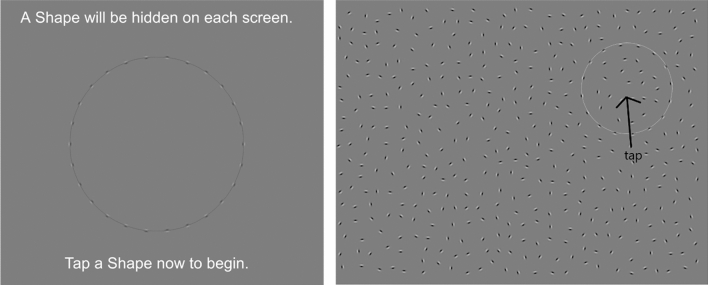
Contour integration task. Gabors appeared on a gray background. Participants were asked to tap on the circle that was formed by the Gabors. The contour is outlined for illustrational purposes.

In this shape detection task^44^, participants were asked to identify a circle that was hidden amongst a field of randomly oriented Gabors (Fig. 4). The circle was comprised of spaced oriented Gabor inducers, similar to a dotted line drawing. Participants were asked to tap on the circle as quickly as possible. To increase the task difficulty, a 3-down, 1-up staircase was run on the orientation jitter of the inducers (i.e., the offset of their orientation from the true orientation appropriate to properly define the contour) such that the smooth curvature of the circle was broken up by the inducers having systematically increasing errors in local slope. In this way, as the orientation jitter increased, the lines of the circle were harder to follow, and the circle faded into the background that was made of randomly oriented Gabors. There were approximately 40 trials per participant depending on the staircase. The dependent variable was the participants’ threshold of orientation jitter of the Gabors (estimated as the average of the last 3 reversals of the staircase).

### Procedure

**Figure 5:**
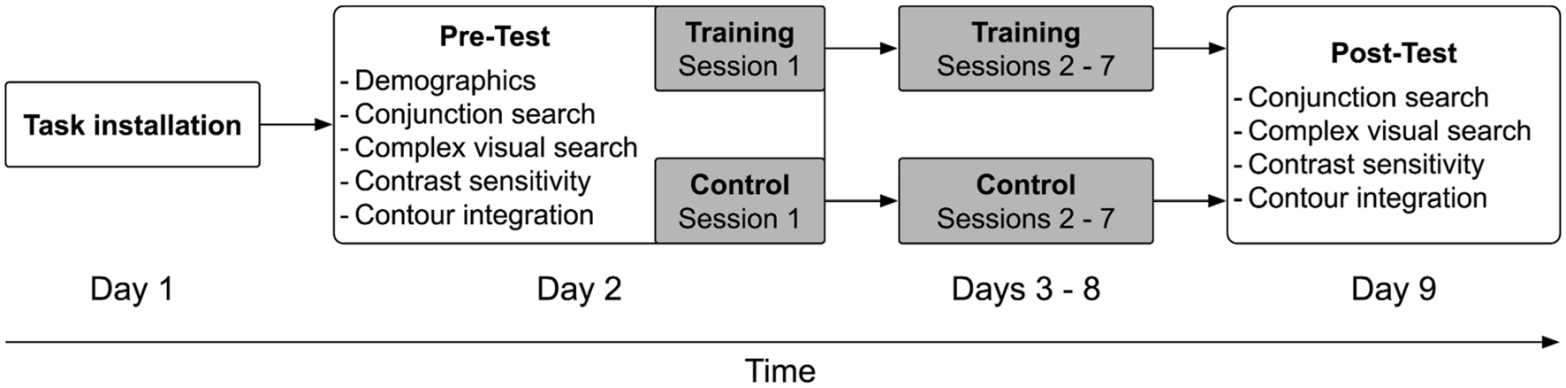
Procedure of the study. Participants were randomly assigned to either the change-detection training group (n = 25) or the active control group (n = 25).

The study was conducted remotely on participants’ own computers. On the first day of the study, all necessary computer tasks were installed under the guidance of research personnel. For that purpose, PsychoPy^45^, Sightseeing^46^, and corresponding experiment scripts were installed. This session lasted about 30 min. Each participant completed a pre-test session consisting of a demographic survey that also included questions related to vision health, a conjunction search task, a complex visual search task, a contrast sensitivity task, and a contour integration task. The pre-test took about 90 min to complete. Following random assignment, participants trained for seven sessions either on the change-detection task or the control task. Participants completed one session per day (excluding weekends) which lasted about 30 min each. The pre-test and the first training session were completed on the same day. Following their last training day, participants completed a post-test session that included the same tasks as the pre-test session. The post-test lasted about 60 min. The study procedure is represented in Fig. 5. During all pre- and post-test assessments, as well as during training, participants were supervised via video conferencing software that allowed us to monitor compliance and answer any potential questions participants might have; a procedure that has shown to result in similar data quality and performance as compared to in-lab studies^47^.

## Results

In our statistical approach, after screening for outliers, we first tested for baseline differences and then for an overall effect of training on all our outcome measures with a multivariate analysis of variance (MANOVA) and an independent samples t-test for group differences on the averaged z-scores of our four outcome measures. Next, we followed-up on these analyses with independent samples t-tests, analyses of covariance (ANCOVAs), and repeated measures analyses of variance (ANOVAs). We report and mainly rely on frequentist analyses, but we also provide Bayes Factors using default priors to provide a more comprehensive analysis picture. The study procedures and analytic approaches were pre-registered at AsPredicted (Protocol #46326).

### Training outlier analyses and evaluation of baseline differences

All analyses were carried out with JASP^48^. To screen for non-compliance and to identify low-performing outliers in the change-detection training data, we analyzed the R^2^ of individual regression models with logarithmic transformations of session number. Outliers were defined as regression models with an R^2^ value that was more than three median absolute deviations below the overall median^49^. This analysis revealed no training outliers in our data. However, there were two participants, including the only one who performed worse at the end of training than in the beginning, with an R^2^ = 0.000. Given that the fit of the average curve across all participants was R^2^ = 0.996, we ran our analyses with and without these two participants. Because there were no substantial differences between both analyses and to avoid the risk of bias, we only report the analysis with the whole sample.

Independent samples t-tests and Mann–Whitney U-tests revealed no significant group differences at pre-test (all *p*s > 0.199; all *BF*_*10*_ < 0.403) in any of our dependent variables. The descriptive statistics of the raw data are provided in Table 1 and the Pearson’s correlations between pre-test measures in Table 2.

**Table 1:** Descriptive data of the outcome measures for both groups.

	Pre-test					Post-test					Pre-post comparison				
	*N*	Mean	SD	Min	Max	*N*	Mean	SD	Min	Max	BF_10_	*t*	*p*	*r*	Cohen's d
**Experimental group**															
Conjunction search (ms)	25	1010	197	685	1469	25	924	182	614	1332	6.735	3.434	0.002	0.776	0.687
Complex visual search (s)	24	73	22	35	127	24	65	15	32	91	1.001	1.901	0.070	0.304	0.388
Contrast sensitivity	25	0.168	0.135	0.033	0.451	25	0.103	0.114	0.012	0.451	4627	9.155	< 0.001	0.916	1.831
Contour integration	20	0.314	0.096	0.083	0.467	20	0.294	0.092	0.153	0.489	0.368	1.021	0.320	0.541	0.228
**Active control group**															
Conjunction search (ms)	25	997	148	750	1396	25	1042	222	715	1529	0.51	− 1.064	0.298	0.687	− 0.213
Complex visual search (s)	24	71	19	32	115	24	72	16	33	100	0.217	− 0.077	0.881	− 0.034	− 0.031
Contrast sensitivity	25	0.150	0.111	0.041	0.387	25	0.095	0.085	0.017	0.332	26,415	12.655	< 0.001	0.948	2.531
Contour integration	19	0.289	0.129	0.039	0.600	19	0.286	0.071	0.178	0.415	0.238	0.095	0.925	0.079	0.022

**Table 2:** Pearson's correlations between all four outcome measures at pre-test.

	Conjunction search	Complex visual search	Contrast sensitivity
Conjunction search	–		
Complex visual search	− 0.040	–	
Contrast sensitivity	0.261	0.044	–
Contour integration	− 0.161	0.021	− 0.496**

**p < 0.01.

### Training performance

**Figure 6:**
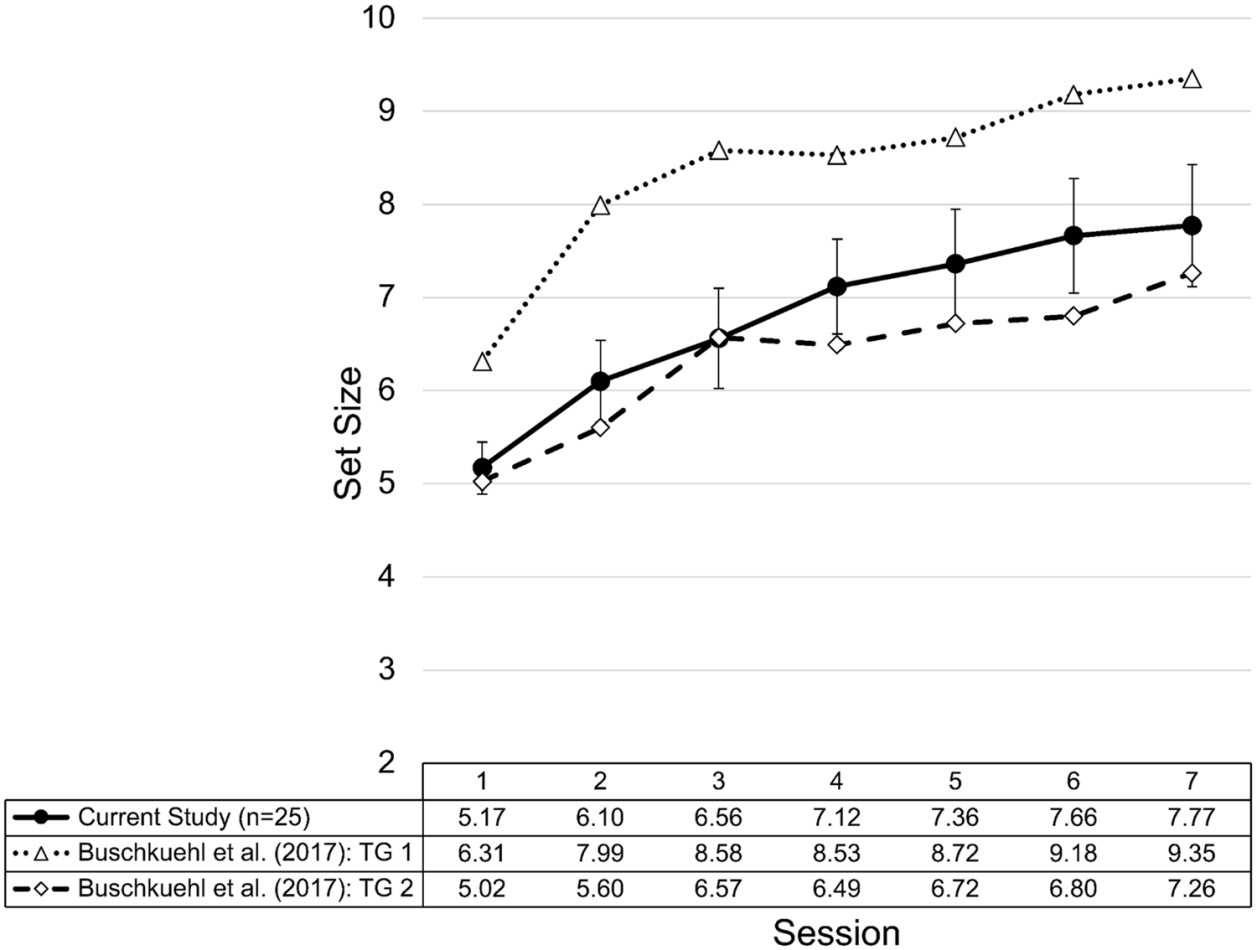
Training curve of the current study compared to the first seven training sessions of the two training groups reported in Buschkuehl et al.^7^. Despite some conceptual task differences, training performance of the current study is similar to that of Training Group 2 (TG2) from Buschkuehl et al.^7^, however, the overall performance is descriptively higher throughout most of the training sessions. Error bars represent standard error of the mean. TG1 stands for ‘Training Group 1’.

Participants in the experimental group increased their performance by 50% over the course of seven training days (Fig. 6). A one-way (training sessions: 1–7) repeated measures ANOVA revealed a significant main effect of session, *F*(2.856,144) = 19.767, *p* < 0.001, *η*^2^_p_ = 0.452, *BF*_*10*_ = 5.288e + 13. A regression analysis with logarithmic transformations of session number revealed an excellent fit (R^2^ = 0.996) that was better than the fit of a linear regression model (R^2^ = 0.927).

Participants in the control group demonstrated a performance increase of 34% from the first to the last session. A one-way (training sessions: 1–7) repeated measures ANOVA revealed a significant main effect of session, *F*(6,144) = 10.095, *p* < 0.001, *η*^2^_p_  = 0.296, *BF*_*10*_ = 3.977e + 6.

### Outcome variables

#### Outlier control

##### Conjunction search task

We screened for outliers and deleted all data points that were more than three median absolute deviations above or below the overall median^49^. This screening was conducted separately within each participant, for each set size (5, 10, 15, and 20), and both sessions (pre- and post-test). It resulted in the removal of 259 out of 3948 data points (6.6%). We then averaged the reaction times for each set size separately. For the purpose of the main analysis, the mean reaction times for each set size were averaged again, resulting in a single performance metric for each participant, from which we then calculated the gain scores. We also note that participants made on average fewer than 2 errors per test occasion.

##### Complex visual search task

We excluded two participants (1 experimental, 1 control) from the analysis because they did not find the target in any of the 12 search images presented to them at pre-test. Overall, we had eight participants (four in each group) at pre-test and four participants at post-test (2 in each group) who found fewer than four out of the 12 targets. Nevertheless, Cronbach’s α on the accuracy scores was 0.735 at pre-test and 0.695 at post-test. Due to the variation of inherent difficulty between the search images, it did not make sense to control for outliers and therefore mean reaction times were created for each participant, which served as the basis for the gain score calculation.

##### Contour integration task

We had to exclude a total of 11 participants (5 experimental, 6 controls), 7 (3 experimental, 4 controls) due to insufficient screen resolution that was required to run the task, and 4 (2 experimental, 2 controls) due to non-compliance with instructions.

Finally, in order to fit the data of the conjunction search task and the contrast sensitivity task better to a normal distribution, the corresponding pre- and post-test data were log-transformed before calculating gain scores.

#### Overall analysis

We conducted a MANOVA to investigate whether change-detection training resulted in an overall improvement in visual processing. For that purpose, we generated gain scores (pre-test minus post-test) for all four of our outcome measures that served as the dependent variables; group constituted the between-subjects variable. The overall MANOVA was significant, *F*(4,34) = 3.051, *p* = 0.030, *η*^2^_p_  = 0.27. Following-up on the MANOVA, independent samples t-tests on the gain scores yielded a significant group effect for the conjunction search task, *t*(37) = 3.316, *p* = 0.002, *d* = 1.062, *BF*_*10*_ = 17.339, but not the other tasks: complex visual search, *t*(37) = 1.017, *p* = 0.316 *d* = 0.326, *BF*_*10*_ = 0.469; contrast sensitivity, *t*(37) = 1.313, *p* = 0.197, *d* = 0.421, *BF*_*10*_ = 0.613; contour integration, *t*(37) = 0.461, *p* = 0.648, *d* = 0.148, *BF*_*10*_ = 0.339.

**Figure 7:**
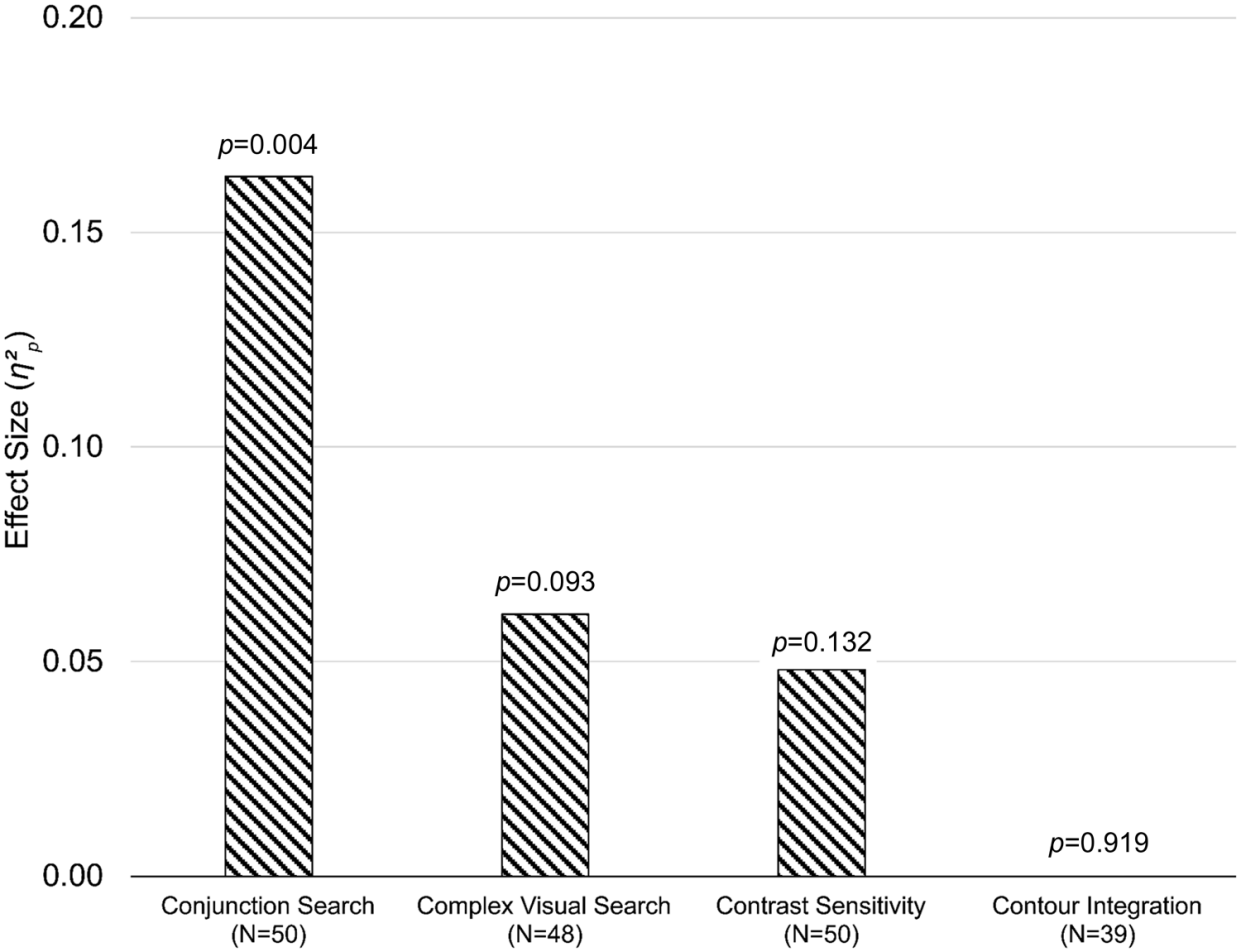
Effect sizes for each outcome measure. The effect sizes are based on individual ANCOVAs and the p-values above each bar represent the significance level of the corresponding analysis.

An issue with the MANOVA was that the analytical sample was reduced to N = 37 (excluding 6 participants from the experimental group and 7 controls) due to missing data, especially in the contour integration task (11 missing data points) but also the complex visual search task (2 missing data points). Therefore, in order to increase the statistical power by including all available data, we z-scored all gain scores that constituted the dependent variables of the MANOVA and averaged these z-scores across individual participants. An independent samples t-test revealed a significant group effect, *t*(48) = 2.742, *p* = 0.009, *d* = 0.776, *BF*_*10*_ = 5.471. To further analyze the data of the four outcome measures, we ran separate ANCOVAs with the post-test scores as the dependent variable and the pre-test scores as the covariate. These analyses considered all available data points per outcome measure and revealed a significant group effect for the conjunction search task, *F*(1,47) = 9.179, *p* = 0.004, *η*^2^_p_  = 0.163, *BF*_*incl*_ = 9.348, but not the other tasks: complex visual search, *F*(1,45) = 2.941, *p* = 0.093, *η*^2^_p_  = 0.061, *BF*_*incl*_ = 0.895; contrast sensitivity, *F*(1,47) = 2.351, *p* = 0.132, *η*^2^_p_  = 0.048, *BF*_*incl*_ = 0.708; contour integration, *F*(1,36) = 0.010, *p* = 0.919, *η*^2^_p_  = 0.000, *BF*_*incl*_ = 0.319. We note that the significance levels of this set of ANCOVAs do not change if the p-values are adjusted for multiple comparisons, for example through a Bonferroni correction. Corresponding repeated-measure ANOVAs led to nearly identical results and are therefore not reported. Figure 7 provides an illustration of the effect sizes for each of the four outcome measures.

#### Detailed conjunction search task analysis

**Figure 8:**
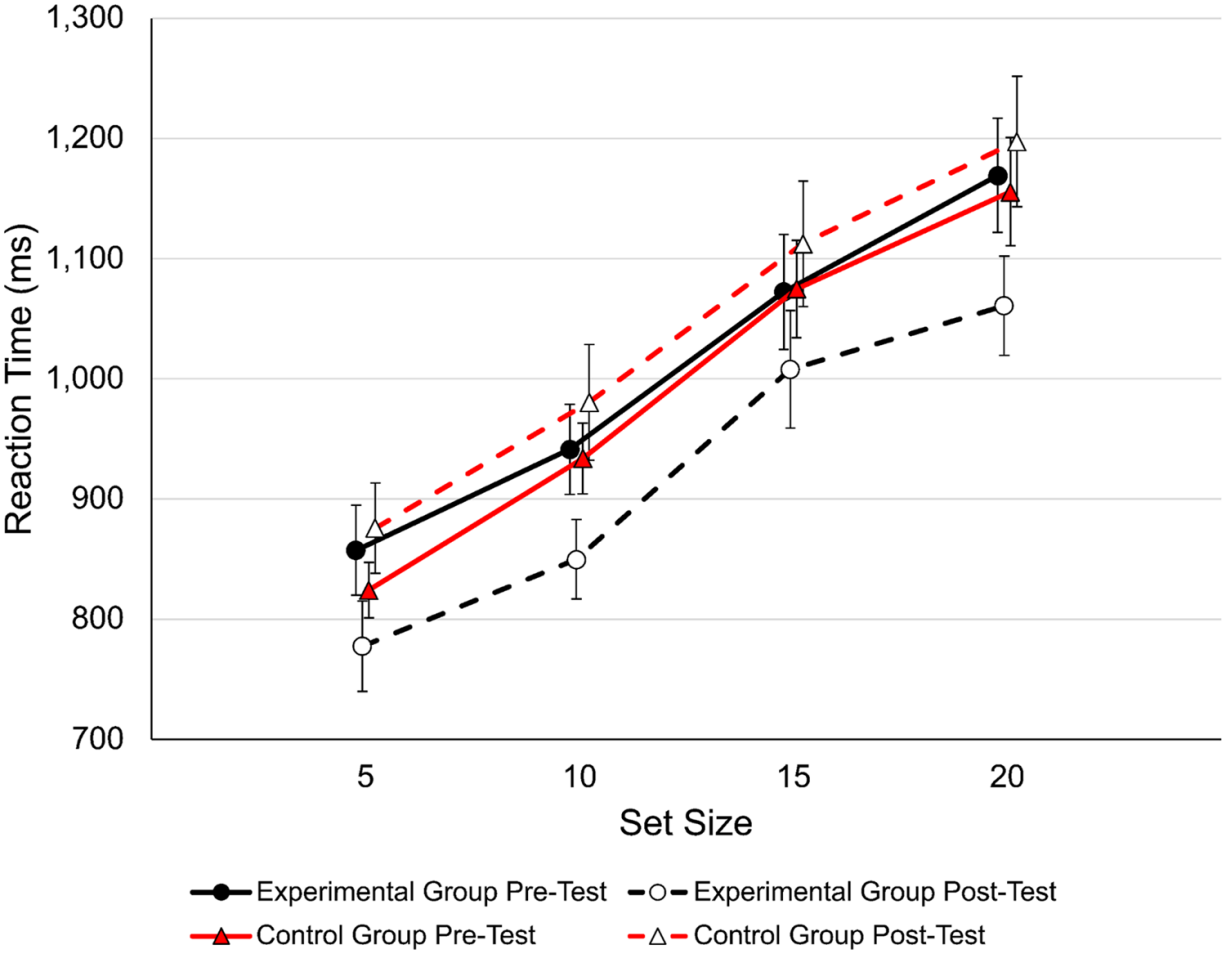
Reaction time data of the conjunction search task. The data show the typical slope that indicates how much longer it takes to find a target with an increasing number of distractors. While both groups performed similarly at pre-test, the experimental group improved at post-test and the control group got worse, although to a smaller degree. The averaged slope of fitted linear regression models to the actual reaction times at pre-test resulted in a value of 21.26 ms (SE = 1.40) which is well in line with previously reported data^40^. Error bars represent standard error of the mean.

To further elucidate the outcome of the conjunction search task, we analyzed the reaction time data as a function of set size (Fig. 8). The basis for our reaction time analysis was the outlier-controlled data, averaged for each set size. To improve the fit of the data to a normal distribution, all means were log transformed. A repeated measures ANOVA with the factors group (experimental vs control), session (pre vs post), and set size (5, 10, 15, 20) revealed a significant main effect for set size, *F*(3,144) = 202.488, *p* < 0.001, *η*^2^_p_  = 0.808, *BF*_*incl*_ = 7.967e + 56, and importantly, a significant session by group interaction, *F*(1,144) = 9.408, *p* = 0.004, *η*^2^_p_  = 0.164, *BF*_*incl*_ = 59,348. There was no main effect for session *F*(1,144) = 2.262, *p* = 0.139, *η*^2^_p_  = 0.045, *BF*_*incl*_ = 2.137 and no main effect for group *F*(1,48) = 1.228, *p* = 0.273, *η*^2^_p_  = 0.025, *BF*_*incl*_ = 0.568. Bonferroni corrected post-hoc tests comparing the effects of set size revealed that all comparisons were significant (all *p*s < 0.001, all *d*s > 0.683, all *BF*_*10, U*_ > 2328). Note that we report *BF*_*incl*_ according to the suggested method by Sebastiaan Mathôd^50^ where applicable.

As can be seen in Fig. 8, the session by group interaction was driven by the improvement of the experimental group, but it is also the case that the control group got worse at post-test, although to a smaller degree. To quantify any group differences of the reaction time x set size functions, we fitted linear regression models for each participant to the mean log-transformed reaction times. ANCOVAs with the post-test scores as dependent variables and the pre-test scores as covariates yielded a significant group effect for the intercept, *F*(1,47) = 4.057, *p* < 0.05, *η*^2^_p_  = 0.079, *BF*_*incl*_ = 1.379; but not the slope, *F*(1,47) = 0.046, *p* = 0.832, *η*^2^_p_  = 0.000, *BF*_*incl*_ = 0.287.

## Discussion

The present study aimed to explore the potential benefits of change-detection training on the perceptual processes of visual search, contrast sensitivity, and contour integration. After seven days of training, participants improved on their initial change-detection performance by 50% on average, providing further evidence for the malleability of change-detection skills with adaptive training. Compared to an active control group, participants also showed generalized improvements across the visual perceptual tasks. However, the improvement was most pronounced in the conjunction search task (*η*^2^_p_  = 0.163). The overall effect was driven to a much lesser extent by the complex visual search task (*η*^2^_p_  = 0.061) and the contrast sensitivity task (*η*^2^_p_  = 0.048). No effect was observed for contour integration (*η*^2^_p_  = 0.000).

### Specific training effects

Although our implemented training paradigm was not identical to the ones used by Buschkuehl et al.^7^, the paradigms are similar enough to warrant a brief comparison. As illustrated in Fig. 6, the training performance observed in the present study is situated between the two training paradigms used by Buschkuehl et al.^7^, which may be due to the fact that the current change-detection training represents a “mix-and-match” of Training Group 1 (TG1) and Training Group 2 (TG2). The current change-detection training task adopted the mask from TG2 and the whole array probe from TG1. The element that notably differentiates the current change-detection design from TG2 is the test display which likely contributes to the better overall training performance. It has been found that such cues lead to improved change-detection performance as they assist in retrieval of memory representations^51,52^. In addition, configurational grouping may have been a strategy used by participants to result in better performance^7^. By possibly grouping nearby squares together, participants may have an easier time recalling the color of the squares as they are now part of “smaller wholes” instead of being perceived or remembered as “many individual objects”^53^. Another area in which the present training paradigm differs from the ones used previously is training time. The current training task consisted of 20 rounds with each round being composed of 20 trials. In contrast, previously we included only 15 rounds, each round consisting of 20 trials but trained for 10 days instead of only 7. However, the trial lengths were quite similar across the three paradigms. The additional 25% of training time in the first seven sessions in the present study could contribute to the explanation why overall training performance was better here.

### Outcome measures

The main question we aimed to answer was whether change-detection training impacts visual perceptual processes beyond the trained task. At a global level, our data indicate that this is indeed the case, however, the degree of improvement varied substantially at the individual task level. The strongest support for a causal relationship between change-detection training and improved visual perceptual processes comes from the conjunction search task (*η*^2^_p_  = 0.163). Performance on the conjunction search task is captured by a baseline processing time (often referred to as the intercept of a linear regression model) and the rate of processing (often referred to as the slope of a linear regression model). While the rate of processing is an indicator for search efficiency that reflects the rate of attentional shifting between items for example, the baseline processing time is assumed to represent components that are not search related such as perceptual processes and motor-related response processes^54,55^. Figure 8 illustrates that the baseline processing time is different between sessions and groups which was confirmed by a significant group difference of the intercept as shown with the ANCOVA. However, participants processed items at a similar rate before and after training (i.e., all four curves are parallel) as indicated by a non-significant group comparison of the slopes. Therefore, it seems that change-detection training facilitates perceptual processes that are required in our conjunction visual search task. If the training resulted mainly in improved post-search processes, such as motor responses, or a general improvement solely because participants were part of the experimental group, then we would expect to find a more universal improvement across outcome measures, a pattern that we neither observed in the present nor our previous training study^7^. Joseph et al.^54^ have argued that it is hard to distinguish between perceptual and attentional processes using baseline processing and the rate of processing. However, we note that the data of our conjunction search task are strikingly clear in that the only pre- to post-test change was in baseline processing, suggesting that what participants seem to mainly improve on are in fact perceptual processes. This raises the question how much of the observed improvement in the training task can be attributed to improved (task-specific) memory capacity and how much can be attributed to improved perceptual processes. The currently available change-detection training literature suggests that there is negligible transfer potential to tasks that also rely on memory capacity but involve perceptual processes to a far lesser extent, such as an n-back task or a complex span task^2,7,8,10^. This pattern of findings in the literature along with the current outcome suggest that change-detection training seems to most likely improve perceptual processes, but future studies are needed to further clarify this issue. Future research also needs to clarify under what perceptual conditions a transfer from change-detection training to a conjunction search task might occur. For example, the change-detection training task used here covers a relatively wide area of 12.66° by 9.5° of visual angle, and therefore, it is reasonable to assume that participants learn to rely on peripheral cues^56^. A straight-forward way to investigate this assumption would be to parametrically vary the size of the visual field in the conjunction search task.

Although the effect size of the complex visual search task (*η*^2^_p_  = 0.061) indicates that the task contributed to the overall effect of change-detection training on visual processing to some extent, the individual task analysis did not reach significance. We included this task assuming that it differed to a higher degree relative to the conjunction search task from the experimental training task, such that the lack of task similarity would only minimally foster the applicability of rules acquired through perceptual learning^36^. In retrospect, however, we identified a few issues with the task variant we used here, in particular, we think that only having 12 trials in the pre- and post-test session, respectively, was not optimal as only 20 participants were able to find more than half of the targets in the pre- and post-test, respectively. Another issue we identified was that the search images varied in size as a function of the different screen sizes on which the task was run because participants performed the training as well as the outcome measures on their own hardware. The issue is not severe within-participants, but more so on a between-participants level because on larger screens it is intuitively easier to find the targets compared to smaller screens. For that reason, it is important to replicate our initial data in the laboratory using a more controlled environment. Aside from these affordances for methodological improvements, we note that the lack of transfer is in line with our prediction: the complex search task differs from the conjunction search task in that the search images did not consist of discrete items but rather a very complex scene containing many different stimuli. Therefore, it is possible that participants learned to work with highly artificial stimuli such as the squares in the change-detection task, and thus, they were able to apply their learned skills to the orange T stimuli in the conjunction search task but not beyond this specific context. The fact that the initial performance in the conjunction task was relatively low could have further pronounced the effects.

The effect size of the contrast sensitivity task (*η*^2^_p_  = 0.048) was comparable with the one of the complex visual search task (*η*^2^ = 0.061). We note that extensive training is generally required to find robust changes in contrast sensitivity^57^, with typical vision training studies targeting contrast sensitivity employing 20 training sessions^17,58–60^. Still, we note that there were notable improvements in both groups, which is not unusual as there are also known practice effects especially in perceptual or attention-based tasks when comparing two initial subsequent task sessions^61,62^. While some may question why change-detection training would lead to changes on contrast discrimination, which is typically considered a low-level visual feature, one explanation could be on the extent to which attention is focused on luminance properties of stimuli in both the change-detection and the contrast sensitivity tasks. Further, while classical studies have assumed that perceptual learning is driven by low-level processes, it is clear that learning can occur at a variety of levels of processing^12^ and that potential transfer can depend upon on how applicable newly learned rules (e.g., attend to the luminance distribution of a stimulus) are to new situations^36^. Future studies need to be conducted to further quantify the effects of change-detection training and whether longer training would lead to larger effect sizes and more specifically what brain structures, or rule processes, lead to transfer between the tasks.

Compared to the three other outcome measures, there was no effect of training on contour integration (*η*^2^_p_  = 0.000). We speculate that the change-detection training task does not provide enough affordances to improve on this specific perceptual skill. A striking difference between our training task and the contour integration task was that while the training relied on straight lines of its stimuli, the outcome measure focused on circles. Since contour integration performance deteriorates for contours that become increasingly curved compared to contours that are straight or contain corners, the level of difficulty of our contour integration might have been too high to detect any changes as a function of training^63,64^. It is also possible that the focus on individual features that was trained in the change-detection task was inappropriate to lead to positive changes in integrating multiple features into wholes as needed in the contour integration task. Finally, our contour integration task allowed participants to respond within a relatively wide time window which further increases task difficulty, as it has been shown that the ability to detect a contour worsens with prolonged presentation^31^.

### Potential mechanisms of change-detection training

The change-detection task is often used as a measure to assess working memory capacity and some have argued that it is especially well suited to do so because certain strategies such as rehearsal are only minimally helpful due to the fast timing of the task^1^. It has also been argued that change-detection performance represents a fairly stable trait^5^. Nevertheless, a growing number of training studies using change-detection tasks, including the current experiment, demonstrated that participants can improve on the task to an impressive degree if the training is implemented in a certain way, for example by adaptively adjusting the number of stimuli participants train with. The question then is, what do participants actually learn? While previous training studies suggested that training effects are quite task specific in that there are only very minimal benefits beyond the task context, previous experiments observed an improved fidelity of internal memory representations. We have argued earlier that training on change-detection may lead to improved processes related to perceptual learning and predicted such transfer to most likely occur in visual search tasks given the presumed overlap in cognitive processes shared between working memory and visual search^22,23^. While some might find such a transfer not very surprising, our study is nevertheless the first that causally links change-detection performance, a measure traditionally used to assess working memory capacity, with visual search performance. Future research needs to investigate to what extent the variance in the change-detection task might be explained by working memory processes and/or perceptual processes. One way to investigate this further is by means of mediation models^65^. Critically, this is also an example where cognitive training studies might provide causal insights that correlational studies are not able to provide.

### Limitations

Our entire study, including pre- and post-test assessments, was conducted on participants’ own hardware, which, for example, led to slight variations in stimuli sizes and presentation times. While such studies have been conducted successfully before^47,66^, we would have preferred to better standardize the assessment of our outcome measures by using identical screens and computers across all participants, as well as ensuring that participants completed their sessions in a quiet environment with minimal distractions. Despite those circumstances, our training data were consistent and well in line with findings of our previous study^7^ in which participants also trained on their own hardware at home. Furthermore, the data from the conjunction search task clearly show the signature picture for the reaction time x set size functions one expects based on decades of prior research. In addition, our data also indicate acceptable reliability values in both groups (*r* = 0.776 and *r* = 0.687, respectively). Overall, our results suggest that the data quality for the training task and the conjunction search task was adequate. Taking a closer look at the three other outcome measures, the pre- to post-test reliabilities of the complex visual search task were low in both groups (*r* = 0.304 and *r* = − 0.034, respectively), although internal reliability on accuracy was in an acceptable range for the present purposes (α = 0.735 at pre-test and α = 0.695 at post-test). As discussed before, we believe that a future administration of this or a similar task could benefit from additional trials and a more standardized administration. The pre- to post-test reliability data of the contrast sensitivity task were very high in both groups (*r* = 0.916 and *r* = 0.948, respectively) and we are confident that our task measured this construct appropriately. The reliabilities in the contour integration task, on the other hand, were low (*r* = 0.541 and *r* = 0.079) in both groups. An issue with this task was also that we had to exclude 11 participants due to insufficient hardware specifications or non-compliance with task instructions which indicates that also here, a more standardized task administration would be beneficial.

We also want to note that 78% of all participants were women. However, our gender distribution is comparable to other change-detection training studies. In six different experiments that reported gender information^2,7,9,10^, the average percentage of included women was 76%. There is currently only one study available^8^, where more men than women participated (10 out of 15). Therefore, our sample is in line with the existing change-detection training literature. However, future research is needed to determine whether there might be any gender differences in change-detection training.

These limitations notwithstanding, our training data are very well in line with previous research, and we contend that the data collected with the conjunction search task and the contrast sensitivity task are reliable and robust. However, reliability indices in the complex search task and contour integration task are not ideal which might have prevented adequate assessment of the transfer potential of these tasks, either because of non-standardized task administration, their task design, or a combination of both.

## Conclusion

Our training study revealed impressive specific training effects on a change-detection task as well as transfer effects to perceptual processes. The data indicate a causal relationship between change-detection training and perceptual processes as required in conjunction search tasks. Since the change-detection paradigm is commonly used to assess working memory capacity, future research should focus on how much of its variance is explained by memory performance and how much is explained by perceptual processes.

## Data Availability

The datasets used in the current study are available from the corresponding authors on reasonable request.
